# The Healthy Minds, Thriving Kids Project: Educator perspectives on relevance and potential impact of a mental health skill building program

**DOI:** 10.1371/journal.pone.0305450

**Published:** 2025-03-24

**Authors:** David Anderson, Jeffrey Chapman, Janine Domingues, Gabriella Bobadilla, Mimi Corcoran, Harold Koplewicz

**Affiliations:** Child Mind Institute, New York, New York, United States of America; University of Cambridge, UNITED KINGDOM OF GREAT BRITAIN AND NORTHERN IRELAND

## Abstract

**Background:**

Healthy Minds Thriving Kids (HMTK) is a free to user mental health skill building program developed by the Child Mind Institute with the aim to normalize conversations about emotional health and provide educators with wellness tools. The aim of this study was to explore the applicability of the HMTK program for universal school-based delivery from the perspective of educators, specifically to understand acceptability of program materials, perception of the quality of the program, and impressions of the program’s usefulness and relevance across K-12 settings.

**Methods:**

The HMTK program was available to view by educator registrants between 01/26/2022 and 09/07/2022 in the State of California. Educator participants viewed an introductory video for the program and a minimum of two skills videos before participating in an online survey.

**Results:**

Of 68,861 registrants to the website, 64,376 provided survey data. Post-pandemic levels of stress and anxiety were increased, and 89.5% of respondents said young people required a greater degree of support than in the past. Almost all educators (90%) endorsed a need for additional mental health skill building tools for students, and following review of HMTK, > 80% of respondents said they would use the program in their classrooms. Most (86.6%) found the program engaging, and 85.1% found the program relevant to and representative of their student cohorts. More than three quarters (79.6%) said their students would find the program engaging and beneficial. Post-exposure to HMTK, 18.8% more educators believed that the State of California was committed to supporting students’ emotional learning.

**Conclusion:**

This survey demonstrates that from the perspective of educators the HMTK program is a valuable and complementary resource to school curricula to improve the mental health skills of young people. It provides an easy-to-implement framework that school districts and administrators can integrate within their curriculum planning.

## Introduction

Childhood and adolescence present a time of immense change for the developing brain [1] and are associated with increases in risky behavior, expression of strong emotions, and impulsivity. [2] Adolescents with mental health conditions are particularly vulnerable to social exclusion, discrimination, and stigma, which affect their readiness to seek help, leading to behavioral or educational difficulties, risk-taking behaviors, and physical ill-health [3]. Mental health among children and adolescents in the USA and Europe has been declining for some time [4,5], and depression, anxiety, and behavioral/conduct problems are prevalent and increasing. The extent of mental health issues among young people is well-validated. By the age of 24 the lifetime prevalence of mental health disorders is 75% [6], with 50% in place by the age of 14 years.

In the context of this landscape of pre-existing mental health decline, the SARS-CoV2 (COVID-19) pandemic has had a significant impact on the emotional health of both young people and adults, increasing reports of stress, anxiety, and depression by up to 25% [7–13]. The effects of COVID-19 on young people’s mental health has been varied, impacting readiness to learn and a range of other factors [9,14–20]. These factors include societal disruption and separation from friends and family, with a pronounced impact on finances, housing, social support, relationships, and daily routines [18–21]. Stay-at-home orders led to pandemic-related job losses or a loss of income until a return to work was possible, increasing instability experienced by young people and their families [17]. Throughout the pandemic, worry about the lethal consequences of COVID-19 among young people increased social isolation [21,22]. Similarly, grief and trauma due to loss of a family member or caregiver due to COVID-19 will have a life-long effect on the thousands of young people who experienced these events [19,23]. Post-COVID, it is clear across many countries that increases in abuse, neglect, violence, or discrimination have occurred as a result of COVID-19. It is suggested this may be as a result of social isolation, lack of interaction with structural support services such as child welfare, and caregivers becoming overwhelmed by stressors in their lives [15].

Whilst addressing deteriorating emotional health is considered a priority for intervention, treatment gaps remain in the USA, particularly for anxiety and behavioral problems [24]. Gaps may be apparent for range of reasons including, but not limited to, fair and equitable reimbursement from insurers for behavioral health treatment [25], lack of funding particularly within the education system to explore and optimize health literacy [26,27], demand for child and adolescent psychiatric services outstripping the supply available [28], and a need to integrate child and adolescent services within the wider landscape of health, familial, educational, or legal contexts. Improving emotional health among children and adolescents must be a national and global priority from an early age and has led to the development of the Healthy Minds, Thriving Kids (HMTK) program.

Furthermore, the World Health Organization (WHO) has indicated that countries need functional, universal mental health promotion and prevention programs [29]. Universal school-based programs to address and improve mental health skills in children and adolescents have demonstrated variable outcomes [30]. Some meta-analyses and studies suggest that universal educational settings or school-based programs improve mental health literacy and build resilience while others suggest improvements may be small or nonsignificant [31–35]. Because of the mixed results in the area of universal prevention, programs like HMTK, grounded in well-established evidence-based concepts in youth mental health, have the potential to bridge these gaps and deliver more reliable improvements in mental health skills*.* HMTK is an innovative mental health skill building program developed by the Child Mind Institute, a nonprofit organization dedicated to supporting children and their families with mental health and learning disorders.

Educators are confronted with a fast-growing number of young people experiencing mental health distress, which has been exacerbated by the pandemic [36,37]. With mental health services for young people under strain, there is increasing pressure on schools and educators to support young people. Across the USA, educators report that they do not receive sufficient training to be able to provide mental health support to their students, a fact that has been established by the National Center for Education Statistics exhorting strategy development at local, state and national levels (https://nces.ed.gov/surveys/spp/results.asp#mentalhealth-march24-chart7) [38]. The HMTK program was developed to provide educators with a child and adolescent mental health skill building framework that would complement existing social and emotional learning programs while also teaching unique, evidence-based skills. The development of HMTK was predicated on the principle that taking a coordinated and evidence-based approach to mental health skill building in children and adolescents in school can improve their outcomes. The mental health skills taught within the HMTK curriculum are drawn from research on active components of evidence-based child and adolescent interventions for young people, with a focus on effective teaching and practice of skills (e.g., emotion identification and monitoring, relaxation skills, etc.) that might be introduced during the initial stages of a course of cognitive-behavioral treatment (CBT) [39–42].

Specific aims of HMTK are to normalize conversations about coping and emotional health and provide educators with skill promoting tools. The program is free of charge to end users to teach skills that children and adolescents can use throughout their whole lives. Program material is based on well-established concepts and techniques in CBT. CBT is well established as a treatment for a wide range of mental health conditions in young people [43]. It is commonly used to treat anxiety and depression and can be useful for a range of other mental health presentations. It is considered the gold-standard evidence-based approach in mental health treatment, based on the concept that the interconnected nature of thoughts, feelings, physical sensations, and actions allows patients to identify and modify specific patterns of thinking and behavior to improve emotional health. CBT is skills-focused and data-driven, equipping individuals with tools to address problems and alleviate symptoms. Through CBT, young people gain skills to understand and cope with distressing emotions and to engage in more positive interactions with family and peers [44]. School-based CBT programs have shown reductions in symptoms of depression and anxiety as well as improvement in coping skills [44]. While CBT interventional strategies have demonstrated good efficacy, it is rational to suggest that pre-emptive use of CBT can promote and contribute to overall emotional health. The HMTK program uses bedrock concepts in CBT to promote mental health skill building and contribute to effective coping with stressors and prevention of future negative mental health outcomes.

Topics covered in HMTK include understanding feelings, relaxation skills, understanding thoughts and their impact on feelings and behaviors, management of intense emotions, and mindfulness. The program is available for three school age ranges – elementary, middle, and high school – and in two languages (Spanish and English). All HMTK videos, implementation guides, and worksheets can be accessed via www.childmind.org/healthyminds.

All HMTK project resources and content were developed by clinicians, educators, and staff of the Child Mind Institute, with video resource creation coordinated by the production company M SS NG P ECES. Video resources were developed to be age and culturally appropriate, utilizing distinct creative concepts for elementary and middle/high school videos. Elementary school videos involved whimsical stories set in an imaginary hedgehog land, with each narrative involving a relevant situation for this age range, illustrative examples of characters expressing feelings, utilizing relaxation skills, or practicing mindfulness, and a brief summary of key points to end the video and direct youth to written resources. Middle and high school videos included a wide range of interviews with a diverse group of youth in each age range discussing emotional challenges that they faced and discussing how they utilized the mental health skills being taught in each video. A narrator then provided introductory, interspersed, and concluding scripted commentary highlighting key teaching points for each mental health skill. These distinct creative concepts, focused on relatability for each age range as well as representative diversity that youth can relate to, were utilized based on the expertise of the Child Mind Institute’s partner production company, M SSNG P ECES, in creating youth-facing content as well as reports from mental health advocacy organizations highlighting best practices for youth engagement [45]. Because of the program’s reliance on well-validated cognitive-behavioral strategies drawn from youth mental health research, it was expected that careful attention to age-matched creative concepts, diversity and relatability, and gold-standard scientific principles would provide the best chance of program impact on mental health skill growth.

With the support of the State of California, Governor Gavin Newsom and First Partner Jennifer Siebel Newsom, Secretary Dr Mark Ghaly, and the California Department of Health and Human Services, the program was piloted among California educators. A survey across California of educators registering via online portal to access the HMTK video resources was undertaken to explore the issues they faced at an advanced phase of the pandemic, their general perception of emotional health resources for educators, and the potential applicability and impact of the HMTK project for their students.

The aim of this study was to explore the applicability of the HMTK program for universal school-based delivery by educators, specifically to understand acceptability of program materials, perception of the quality of the program, and impressions of the program’s usefulness and relevance across K-12 settings. This initial study provides a foundation with which follow-up studies of implementation processes and student-level outcomes can be later compared and evaluated.

## Materials and methods

### Survey development

The survey instrument was developed in consultation with a range of stakeholders, and both the educator portal and survey were created and implemented by the Performance Development Group (PDG) and InsiteHub. All respondents were educators in the State of California, enrolled and validated by PDG via a specifically designed educator portal to view the HMTK program materials. All survey questions were developed using a 5-point Likert scale from Strongly Disagree to Strongly Agree. The questions and top-line data can be found in Supporting Information.

### Survey design

Participants accessed the HMTK portal to view HMTK video materials between 01/26/2002 and 09/07/2022. On entry to the portal, participants consented to participate and completed a pre-program survey, viewed an introductory video for the program, and viewed at least two of the five skills videos. Following review of HMTK materials, registrants participated in a follow-up survey to explore their perceptions of the materials and their utility in the classroom. All registrants of the HMTK portal completing the pre-program and post-program surveys, developed by CMI, and administered by PDG, were eligible to receive a financial reward of $100. The HMTK portal was closed at the end of the survey period. The survey data were analyzed using contingency tables that were created using QPSMR version CL 64 2021.2c and data analyzed in Excel (see Supporting Information).

### Ethics and consent

Whilst no industry statutes exist in the USA for the conducting of market research, all questionnaires developed by CMI were compliant with and adherent to the AAPOR Code of Professional Ethics and Practices, which is comparable to counterpart guidelines in Europe. Informed consent was provided via self-selected participation in adherence with AAPOR guidance. All data were further aggregated to retain complete anonymization, and all personal data was collected under AAPOR standards. Generated data only assessed and documented feedback on the materials of the HMTK program and did not include generalizable knowledge such as biomedical or behavioral research or school records. As such, this survey does not meet criteria found in 45 CFR 46.104(d) and is exempt from IRB oversight. IRB exemption was granted on 05/15/2024 (Pro00079335, Advarra IRB registered with OHRP and FDA under IRB#00000971).

## Results

Between 01/26/2002 and 09/07/2022, 72,680 people registered with the HMTK portal to review materials (Table 1). Of these, 72,097 are English speakers and 583 are Spanish speakers. Participants represented 8,443 schools and 990 school districts, and 60,003 registrants completed requirements to receive their financial incentive (82.5%). Original data can be found in the Supporting Information.

**Table 1:** Overview of registrants to the HMTK portal as of October 2022.

Parameter	Number of respondents N (%)
Total registrants	72,680
Schools represented (n)	8,443
School districts represented (n)	990
Number of Spanish-speaking registrants	583 (0.8%)
Number of English-speaking registrants	72,097 (99.2%)
Educational setting	
Kindergarten to Grade 5 (K–5)	40,552 (55.8%)
Grades 6–8	18,672 (25.7%)
Grades 9–12	13,456 (18.5%)

**Fig 1:**
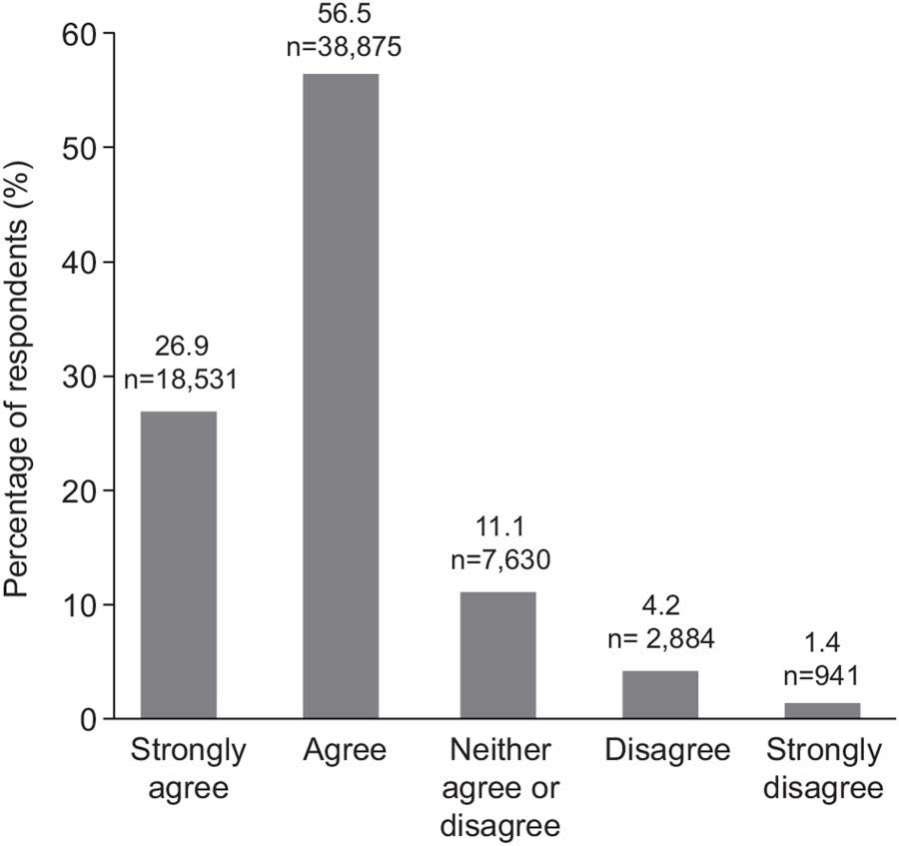
Impact of COVID-19 on mental health and wellbeing. (Q. Since Covid, my students are demonstrating more signs of stress or anxiety in school).

Of registrants to the website, 68,861 opted to take part in the initial survey prior to exposure to HMTK materials, and 64,376 provided data for a survey following their review of HMTK video materials. Almost half of all registrants accessed the program on a desktop computer (48.9%), with 50.6% accessing content on their smartphone and 0.5% accessing via a tablet. Most registrants were Kindergarten to Grade 5 educators (K–5) n = 40,552 (55.8%); 25.7% (n = 18,672) were teaching Grades 6–8, and 18.5% (n = 13,456) were teaching Grades 9–12.

**Fig 2:**
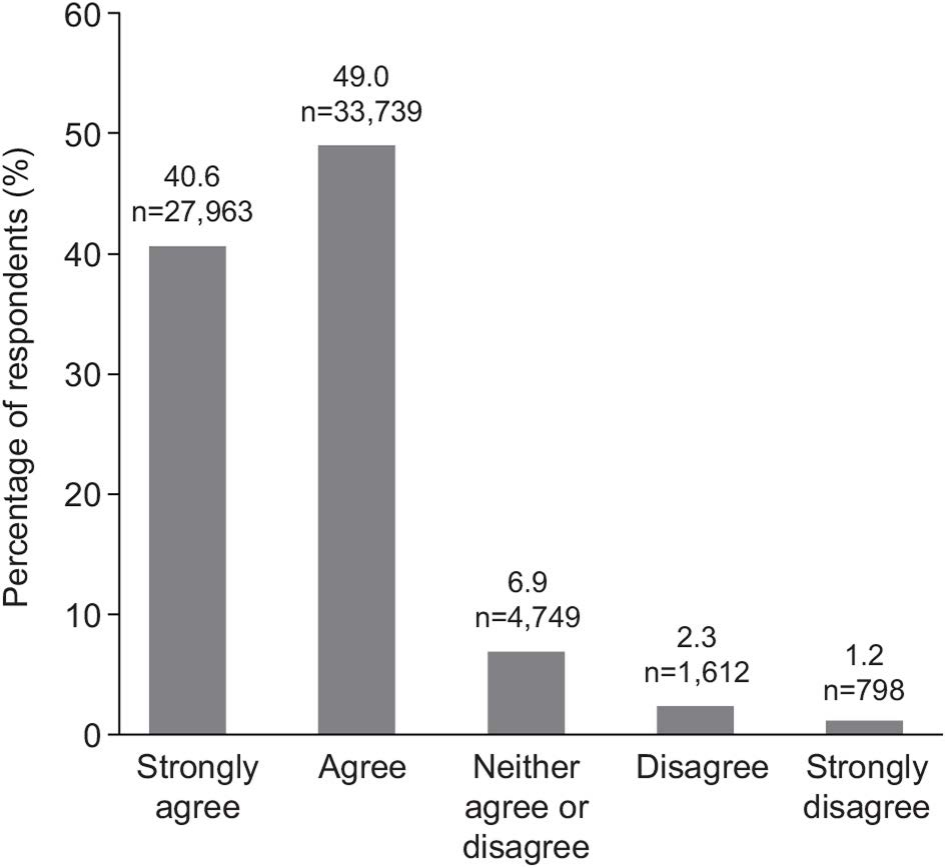
Requirement for increased emotional and social support from educators post-COVID-19. (Q. Since Covid, I am having to support more of my students’ social and emotional learning).

Educator responses related to the impact of COVID-19 on mental health and wellbeing on young people demonstrated high agreement (83.3%) that COVID-19 has heightened stress and anxiety among young people (Fig 1), and results were consistent regardless of the student cohort (Table 2). Similarly, educators indicated that rates of disruptive behavior in the classroom were consistently increased post-COVID-19 across all student age groups. Most educators responded that young people required greater support with respect to social and emotional learning following the pandemic compared with before (89.5%) (Fig 2; Table 3). Most educators said they would benefit from having more social and emotional learning resources for their students (90.2%).

**Table 2:** Impact of COVID-19 on specific educational cohorts. Base n = 68,861.

	Strongly agree N (%)	Agree N (%)	Neither agree or disagree N (%)	Disagree N (%)	Strongly disagree N (%)
K–5 (n = 37,478)	8,650 (23.1)	21,829 (58.2)	4,729 (12.6)	1,798 (4.8)	472 (1.3)
Grade 6–8 (n = 12,609)	3,884 (30.8)	7,049 (55.9)	1,116 (8.9)	408 (3.2)	152 (1.2)
Grade 9–12 (n = 18,774)	5,997 (31.9)	9,997 (53.2)	1,785 (9.5)	678 (3.6)	317 (1.7)

Q. Since Covid, my students are demonstrating more signs of stress or anxiety in school.

**Table 3:** Requirement for support in specific educational cohorts. Base n = 68,861.

	Strongly agree N (%)	Agree N (%)	Neither agree or disagree N (%)	Disagree N (%)	Strongly disagree N (%)
K–5 (n = 37,478)	15,817(42.2)	18,169 (48.5)	2,256 (6.0)	815 (2.2)	421 (1.1)
Grade 6–8 (n = 12,609)	4,629 (42.1)	6,141 (48.7)	792 (6.3)	241 (1.9)	129 (1.0)
Grade 9–12 (n = 18,774)	4,904 (36.4)	9,429 (50.2)	1,701 (9.1)	556 (3.0)	248 (1.3)

Q. Since Covid, I am having to support more of my students’ social and emotional learning.

**Fig 3:**
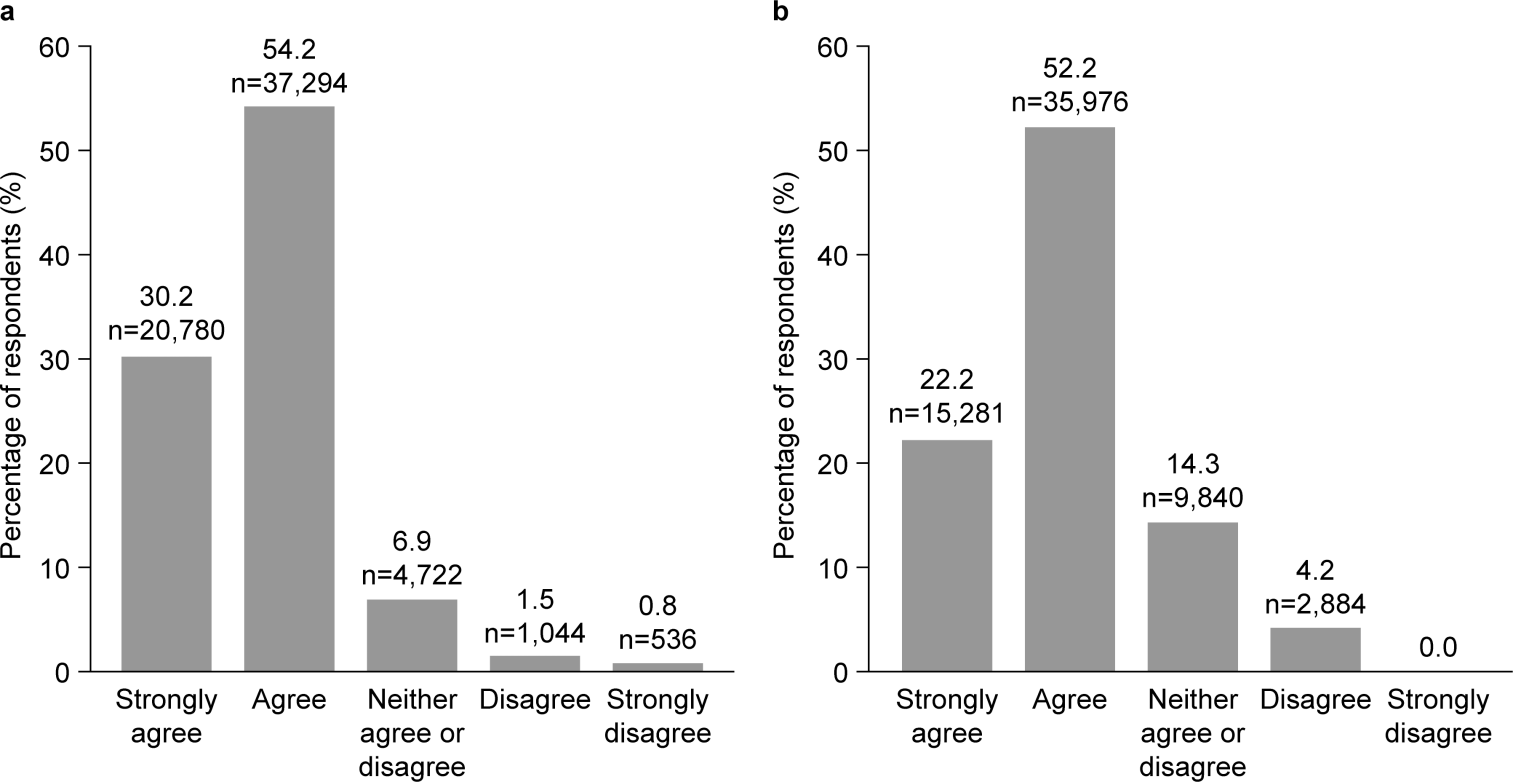
Perception of HMTK by students. (a) My students would benefit from seeing the videos in this program; (b) My students would find these videos interesting to watch.

**Fig 4:**
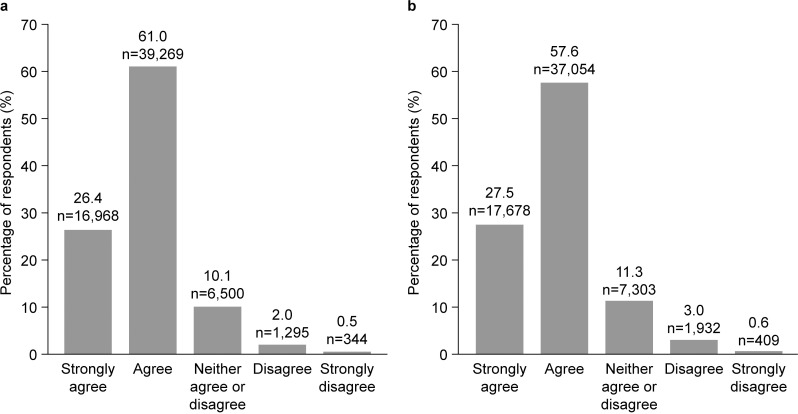
Educator perspectives of the HMTK program. (a) Educator insights into how to approach students’ social and emotional needs; (b) educators’ perspectives of HTMK materials. (Q. (a) I gained insights on how to approach my students’ social and emotional learning needs watching these videos. (b) These videos did a good job representing the diversity of California school students).

Before review of the HMTK video program, almost a quarter of respondents felt that programs to support mental health and wellbeing were not presented in an engaging way, and this was particularly noted in educators teaching Grades 6–8 (19.2%) and Grades 9–12 (17.0%) compared with K–5 (9.4%). Following review of the video materials through the portal, almost all respondents agreed that students would benefit from engaging with the videos within the HMTK program (90.2%), and 79.6% of respondents said they thought their students would find the videos interesting and engaging (Fig 3a, b). More than 80% of educators said the videos provided insights into how to approach students’ social and emotional needs (87.3% (Fig 4a). Educators themselves found the videos engaging (86.6%) and were emotionally moved by the video content (65.7%), and 85.1% felt that the videos were representative of their student cohorts (Fig 4b).

**Fig 5:**
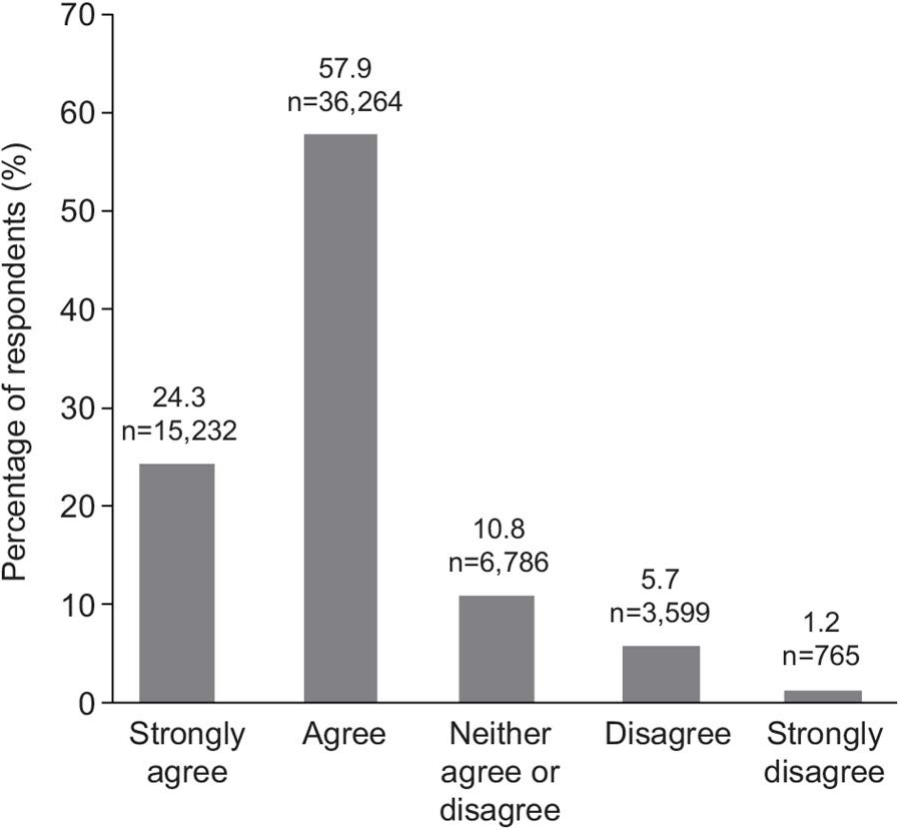
Likelihood that respondents will use the HMTK program as part of their mental health and wellbeing curriculum. (Base n = 62,646. Q. How likely are you to use the videos from this program in your classroom?).

**Fig 6:**
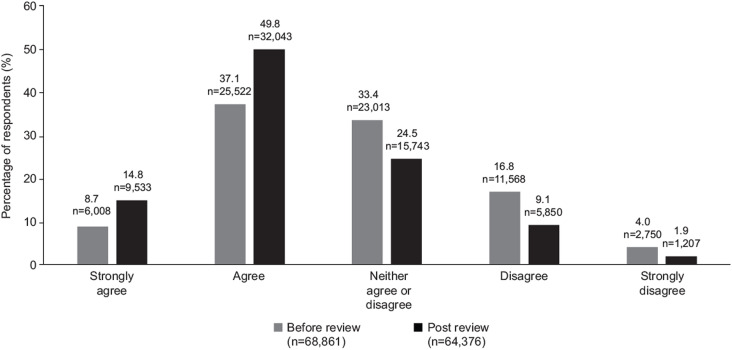
Perceptions of support of educators by the State of California before and after review of the HMTK program. (Pre-review n = 68,861; post-review n = 64,376. Q. I feel the State of California is committed to supporting the social and emotional learning needs of teachers and students).

More than 80% of educators said they were likely to use the videos in their classrooms (82.2%) (Fig 5). When asked if educators believed the State of California was committed to supporting the social and emotional learning of its students, there was a striking increase in agreement between pre- and post-surveys. Before engaging with the HMTK program, only a quarter of educators felt the State of California was committed to supporting students’ social and emotional learning. Following their review of the HMTK video materials, educators demonstrated an 18.8% increase in their belief that the state of California was committed to resources supporting students’ mental health and wellbeing, and only 10% felt that the State of California was not committed (Fig 6).

**Fig 7:**
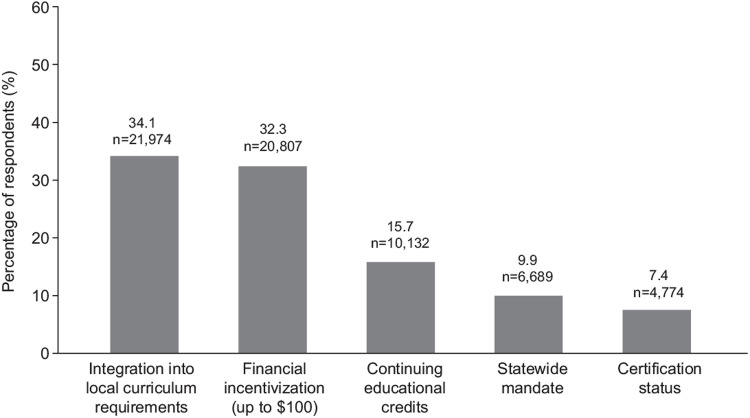
Respondents’ rationale for integrating the HMTK program into their curriculums supporting mental health and wellbeing. (Base n = 64376. Q. What would best motivate teachers like you to use the videos from this program in your classroom?).

When educators were asked following their review of the program what would motivate them to use it in their classroom, 34.1% said that integrating the program into their own existing social emotional curricula was the motivating factor, with 32.3% indicating that financial inducements would incentivize the program’s use. Results were comparable across all student cohorts (Fig 7).

## Discussion

This survey of more than 60,000 educators provides insight into the impact of the COVID-19 pandemic on young people and the needs of educators to build robust and engaging emotional health promotion programs. School is an ideal and critical setting to promote and support the practice of mental health skills. Schools have been identified consistently as the most appropriate setting by policymakers, providing a common setting for all young people regardless of demographic or socioeconomic status [46–48].

Evidence-based mental health prevention programs have demonstrated efficacy in terms of emotional and behavioral problems [49]. Given the evidence indicating the early emergence of mental health and learning disorders during childhood and adolescence, prevention programs focused on building mental health skills among school age young people are incredibly valuable in decreasing the severity of symptom onset while also providing children and teens with the skills they need to cope with stress [6].

Educators recognize that student emotional health is a central part of their students’ readiness to learn, but educators can often feel overwhelmed by the complexity and volume of the issues they are confronted with. They may also feel they lack mental health literacy necessary to address their students’ needs effectively [50–52]. Data from this survey similarly suggests that almost all educators need support materials to help them promote student emotional health. However, given the timing of this survey, it cannot be known how the need for support and resources for educators differs from pre-pandemic needs. This survey has identified a high need among educators for tools in developing learning resources for their students, and survey responses clearly indicate the need for these resources to be engaging and appropriate for the age range they teach. Up to a quarter of respondents suggested that currently available materials were not particularly engaging, especially for older students.

One major advantage of HMTK program materials is the focus on a range of mental health skills, as the program teaches five critical evidence-based coping skills drawn from decades of research on cognitive and behavioral youth mental health interventions. More narrowly focused studies of mental health prevention programs teaching a specific skill such as mindfulness have demonstrated promise [53], but findings are mixed [54]. It may be that prevention programs need to provide a more comprehensive set of skills grounded in a range of evidence-based concepts, moving beyond just mindfulness to explore elements of emotion management, active coping, and understanding thinking patterns. A broader approach helps to build mental health literacy and coping skills among young people accessing the program, and this approach may help to circumvent the effects of individual differences associated with only a narrow range of skills taught.

Survey data suggests that the HMTK video program is a valuable and complementary resource to school curricula to broaden the mental health and coping skill set of young people. More than 80% of respondents endorsed that they had acquired useful strategies from the program that they can take forward into the classroom. Almost all respondents believed the HMTK program would benefit their students and that their students would find these materials interesting and engaging. Respondents demonstrated a high willingness to implement the video program with their students. Encouragingly, the impact of the HMTK video materials was comparable across all educators regardless of the age range of their students, suggesting that the differentiated content of the HMTK program addresses the specific needs of each age group effectively from the educator perspective. However, it must be noted that this first study explored only the applicability, relevance, quality, and usefulness of HMTK program materials. Ongoing follow-up studies exploring implementation processes for the HMTK program and student-level outcomes are needed to explore any nuances of impact of the HMTK program by student group or school setting.

The data from the HMTK portal suggests that from the perspective of educators there is a comparable need to promote and sustain emotional health across all ages of young people from kindergarten to high school. This observation is perhaps surprising given that it might be expected that the impact of COVID-19 lockdown might affect age groups differently, though data in younger children is lacking compared with adolescents [10,18]. In this study exploring the relevance, applicability, and usefulness of the HMTK program from the perspective of educators, no formal inferential testing was done to compare perceptions of educators by more specific demographics or school settings. It will be interesting to see in future studies of HMTK implementation how the efficacy of the program varies by school setting, sociodemographics of students, and educator engagement. Emerging data from our next study exploring HMTK implementation suggests that educators serving students of lower socioeconomic status may be more likely to implement HMTK, but it is not yet known if this is driven by student need or educator motivation. Data from another Child Mind Institute program teaching evidence-based concepts to parents and caregivers suggested that evidence-based information similar to that included in HMTK was even more impactful among certain ethnic minority and LGBTQ + research participants. Comparisons between more specific participant groups in our future studies will undoubtedly provide additional information to inform program adaptation and more targeted implementation support.

Building mental health literacy among educators is important to equip them to provide effective student support. However, implementation of preventative emotional health programs is time consuming and needs to compete with a range of other priorities for educators, along with often limited training and access to resources [50]. It is interesting to note that whilst financial inducements were considered the most powerful incentive by almost a third of respondents, the potential to use the HMTK video program to enhance teaching, linked to the high quality of HMTK resource materials, was equally motivating. Only a small percentage (10.4% overall, 11.5% K–5, 14.0% Grades 6–8, and 8.2% in Grades 9–12) said they would implement the HMTK program only if mandated to, suggesting that respondents recognize the critical need for these materials and value their potential in enhancing students’ academic engagement.

An unexpected element of this survey was the increase in educators’ belief that the State of California is committed to supporting the emotional health of their students. Before review of the HMTK program, 45% of respondents believed that the State of California was committed to supporting them in delivering social emotional learning programs. Post-review of the HMTK program, more than 64% of educators believed the State of California was committed to supporting this need, and the percentage of respondents stating a lack of commitment reduced by half. This suggests that sponsorship of programs like HMTK at the State level can shift public perception and directly empower educators to enhance their support for the emotional health of their students.

With respect to integrating universal school-based mental health skill-building programs, it can be challenging for policy makers to recommend such programs without evidence of positive outcomes. This first study to understand the HMTK program’s applicability to educational settings from the perspective of educators adds practically to our knowledge in promoting student mental health and coping skills. Perhaps this study and those in development have the potential to provide policy makers with confidence to explore and implement universal school-based emotional health programs.

## Strengths and limitations

Whilst the survey cohort was robust, there are several limitations that should be noted. This study of survey questions was only able to track self-reported perceptions and intent to implement rather than surveying actual rates of implementation and outcomes with young people. Understanding the impact of the HMTK program from the perspective of students is an imperative and is the subject of ongoing studies of the HMTK program. Likewise, understanding the impact of the program once it is more widely implemented across more States will be evaluated further with additional studies, and it will be interesting to understand how delivery and implementation are affected by State-specific factors. It will also be compelling in future studies to examine the impact of HMTK resources on students who utilize the program compared against a control group who do not. While data are lacking in the research literature about standard uptake and implementation rates of emotional health programs in school, it is clear that implementation of school-based social and emotional learning (SEL) support is often a multi-faceted, multi-year process with many factors involved affecting educators’ ability to adopt and utilize resources with students.

The strength of this survey is its high number of respondents, providing insight into issues facing educators across California and the challenges their students are experiencing immediately following the COVID pandemic. The HMTK program and its positive reception by educators suggest that these resources can play a critical role in supporting young people, opening up conversations about emotional health and coping skills, and helping K-12 students to navigate the stressors they encounter on the path to adulthood. Companion surveys and studies exploring the impact of the complete HMTK program on educators and its implementation in the classroom are underway. These data will provide even more granular insight into the potential for the HMTK program across California and in other States.

## Conclusion

In conclusion, there is growing evidence of a positive effect on the emotional health of children and adolescents via active implementation of preventative mental health skill building programs. These programs can play a valuable role in building students’ evidence-based coping skills and helping them to cope effectively with mental health challenges. This survey has demonstrated the potential usefulness of a USA-specific skill building program for use across a wide range of educational settings. Studies further demonstrating the impact of this program on the emotional health of young people are underway, with promising indications of the impact of prevention curricula focused on active components drawn from evidence-based interventions for youth and with specific content adaptations for elementary, middle, and high school age ranges.

## Supporting information

S1 File 1. IRB Exemption Statement. **2. Source data:** Educator perspectives on the challenges of emotional health – before review of the HMTK program. **3: Source data:** Educator perspectives after review of the HMTK program
(PDF)
