# Supplementary material for: The Healthy Minds, Thriving Kids Project: Educator perspectives on relevance and potential impact of a mental health skill building program
Source: PLoS One. 2025 Mar 24;20(3):e0305450. doi: 10.1371/journal.pone.0305450 (PMC11932465; doi:10.1371/journal.pone.0305450)

SUPPORTING INFORMATION

The Healthy Minds, Thriving Kids Project: educator perspectives on relevance and potential impact of a mental health skills building program

David Anderson,[1*] Jeffrey Chapman,[1] Janine Domingues,[1] Gabriella Bobadilla,[1] Mimi Corcoran,[1] Harold Koplewicz.[1]

1. David M Anderson, PhD, Child Mind Institute; Jeffrey Chapman, MBA, Child Mind Institute; Janine Domingues, PhD, Child Mind Institute; Gabriella Bobadilla, MA, Child Mind Institute; Mimi Corcoran, MPA, Child Mind Institute; Harold Koplewicz, MD, Child Mind Institute

*Email: david.anderson@childmind.org

EXEMPT DETERMINATION

**DATE:**	15 May 2024
**TO:**	David Anderson, PhD
**PROJECT:**	Child Mind Institute, The Healthy Minds, Thriving Kids Project: A Mental Health and Wellbeing Prevention Program for Young People (Pro00079335)

DOCUMENTATION REVIEWED:

Protocol Version(s):
- Protocol (Not Dated)

Using the Department of Health and Human Services regulations found at 45 CFR 46.104(d) (4), the IRB determined that your research project is exempt from IRB oversight. All study related documents will be removed from our active files and archived.

Note: You will still be able to access this study via the Advarra CIRBI Platform under the "Archived" tab on your Dashboard for three years. After three years, the study will be removed from the system in accordance with IRB regulations.

Please be advised that as Advarra IRB is not overseeing the conduct of the study, study materials, documents and reports should not state that the study is "approved" by an IRB. Also, if your study includes subject-facing materials such as consent forms, recruitment materials, and other materials used by subjects in the study, the IRB company name and contact information should not be referenced. Study materials, documents and reports may include a general statement that the study was reviewed by an IRB, such as, "This study has been reviewed by an institutional review board (IRB), which is a committee that has reviewed this research study to help ensure that your rights and welfare as a research participant are protected and that the research study is carried out in an ethical manner."

The IRB granted this exemption with an understanding of the following:

1. The research project will only be conducted as submitted and presented to the IRB, without additional change in design or scope.

2. Should the nature of the research project, or any aspect of the study, change such that the nature of the study no longer meets the criteria found in 45 CFR 46.104(d) (4), you will resubmit revised materials for IRB review.

3. It is the responsibility of each investigator to ensure that the project meets the ethical standards of the institution. Specifically, the selection of subject is equitable, there are adequate provisions to maintain the confidentiality of any identifiable data collected, and when there are interactions with research subjects,

© Copyright 2023

they will be informed: that the activity involves research; of a description of the procedures; that participation is voluntary; and of the contact information for the researcher.

The IRB will evaluate the new information and make a determination at that time regarding the research project's status.

This project is not subject to requirements for continuing review.

If you wish to appeal the IRB's determinations and/or imposed modifications, please submit supporting documentation to address the IRB's concerns by creating an Appeal Modification in CIRBI.

Compliance Statement/REB Attestation (Applicable for research conducted in Canada):

The IRB attests that this submission has been approved by an IRB whose membership complies with the requirements defined in Health Canada regulations, ICH GCP guidelines, FDA regulations at 21 CFR part 56, and HHS regulations at 45 CFR part 46. The IRB carries out its functions in accordance with FDA regulations at 21 CFR parts 50, 56, 312, and 812; HHS regulations at 45 CFR part 46, subparts A-E; good clinical practices; Health Canada regulations; and the Tri-Council Policy Statement: Ethical Conduct for Research Involving Humans, as appropriate to the research.

Advarra IRB is registered with OHRP and FDA under IRB#00000971.

If you have any questions or concerns, please use the Contact IRB activity on the Advarra CIRBI™ Platform.

Thank you for selecting Advarra IRB to review your research project.

Sincerely,

Luke Gelinas, PhD
Executive Board Chair

© Copyright 2023

Educator perspectives on the challenges of emotional health - before review of HMTK program

Question	# Responses
1 Since Covid, my students are demonstrating more signs of stress or anxiety in school.	68861
Strongly Agree and Agree combined total	57406
percentage	83%

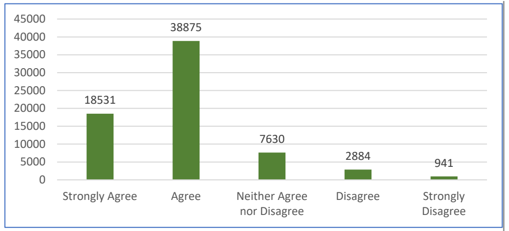

Strongly Agree and Agree combined total		
k-5	6-8	9-12
81%	87%	85%

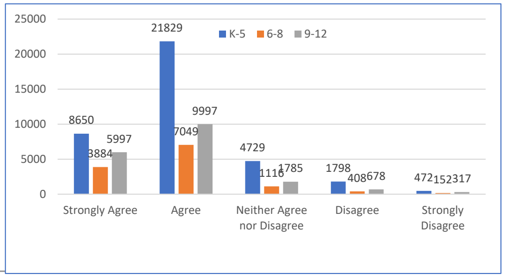

Question	# Responses
2 Since Covid, my students are exhibiting more disruptive behavior in school.	68861
Strongly Agree and Agree combined total	52096
percentage	76%

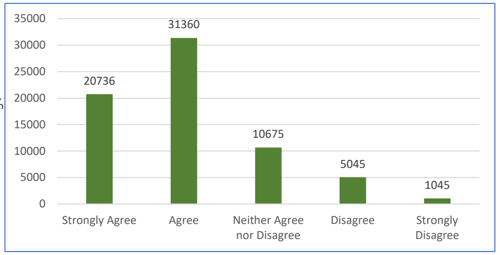

Strongly Agree and Agree combined total		
k-5	6-8	9-12
78%	79%	69%

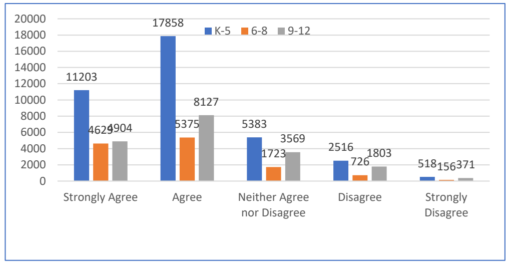

Question	# Responses
3 Since Covid, I am having to support more of my students' social and emotional learning.	68861
Strongly Agree and Agree combined total	61702
percentage	90%

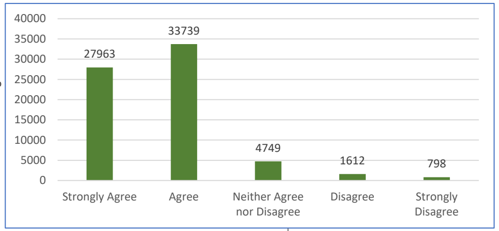

Strongly Agree and Agree combined total		
k-5	6-8	9-12
91%	91%	87%

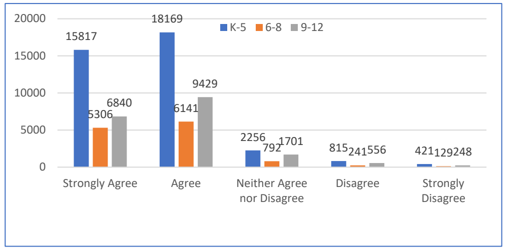

71 32 52

Question	# Responses
4 As a teacher, I would benefit from having more social and emotional learning resources for my students.	68861
Strongly Agree and Agree combined total	62150
percentage	90%

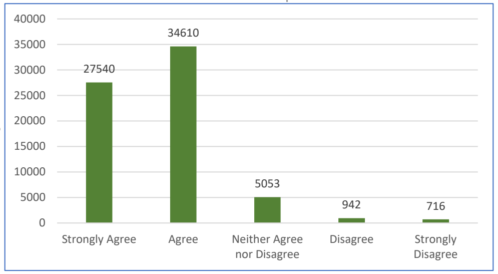

Strongly Agree and Agree combined total		
k-5	6-8	9-12
92%	90%	87%

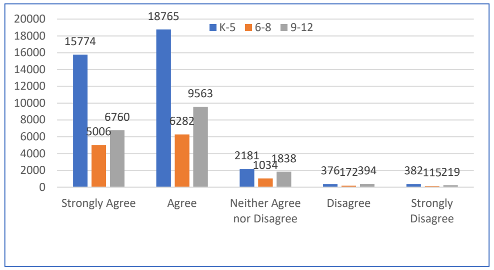

5 Social and emotional learning resources are typically presented in an engaging way.

68861

Strongly Agree and Agree combined total 39510

percentage 57%

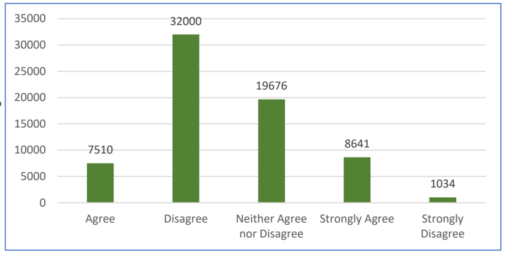

Strongly Agree and Agree combined total

k-5	6-8	9-12
62%	50%	52%

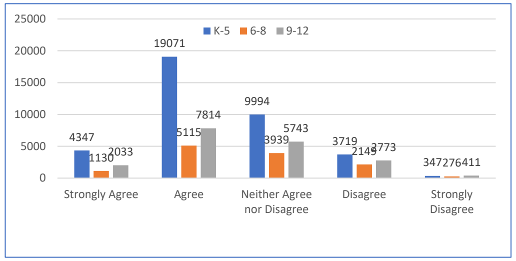

6 I feel the State of California is committed to supporting the social and emotional learning needs of teachers and students.

68861

Strongly Agree and Agree combined total 31530

percentage 46%

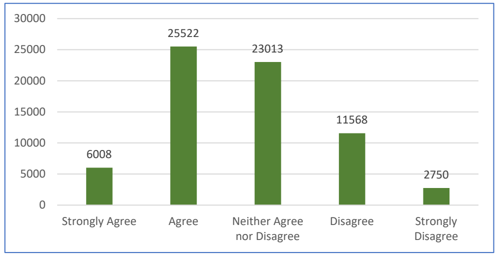

Strongly Agree and Agree combined total

k-5	6-8	9-12
45%	44%	48%

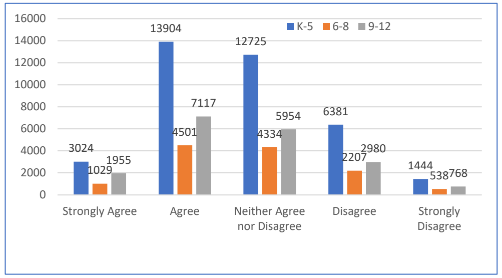

Educator perspectives after review of the HMTK program

	Question	# of Responses
1	My students would benefit from seeing the videos in this program.	64376
	Strongly Agree and Agree combined total	58074
	percentage	90%

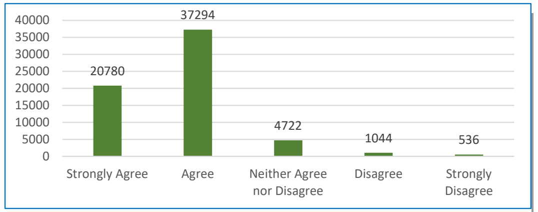

Strongly Agree and Agree combined total

k-5	6-8	9-12
93%	89%	90%

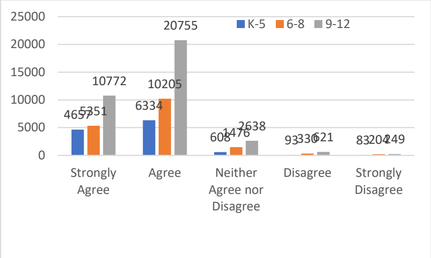

	Question	# of Responses
2	My students would find these videos interesting to watch.	64376
	Strongly Agree and Agree combined total	51259
	percentage	80%

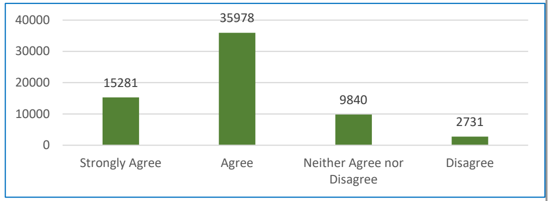

Strongly Agree and Agree combined total

k-5	6-8	9-12
79%	73%	83%

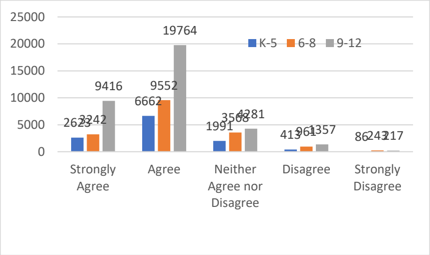

	Question	# of Responses
3	I gained insights on how to approach my students' social and emotional learning needs watching these videos.	64376
	Strongly Agree and Agree combined total	56237
	percentage	87%

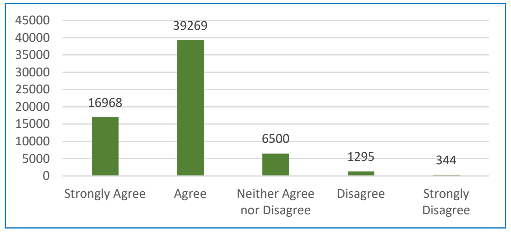

Strongly Agree and Agree combined total

k-5	6-8	9-12
88%	86%	88%

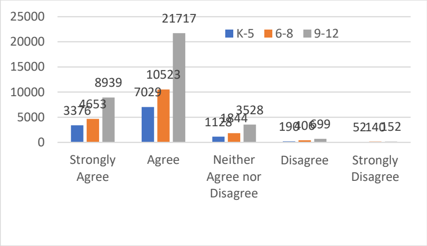

	Question	# of Responses
4	I found these videos to be engaging to watch.	64376
	Strongly Agree and Agree combined total	55796
	percentage	87%

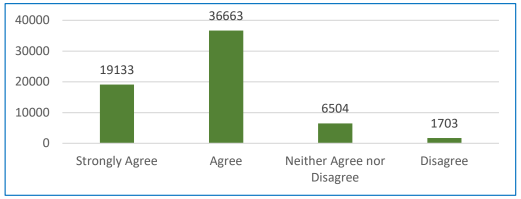

Strongly Agree and Agree combined total

k-5	6-8	9-12
89%	84%	87%

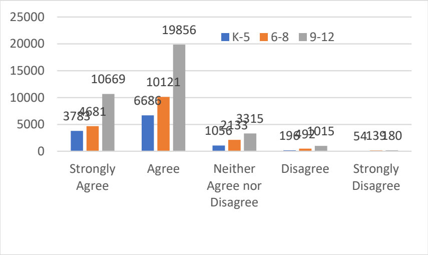

5 I was emotionally moved by these videos. 64376

Strongly Agree and Agree combined total 42345

percentage 66%

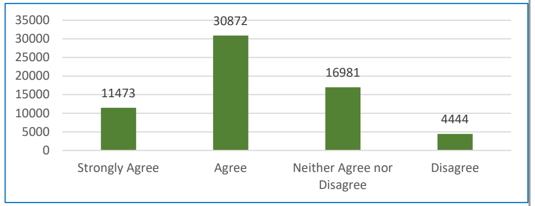

Strongly Agree and Agree combined total

k-5	6-8	9-12
71%	64%	65%

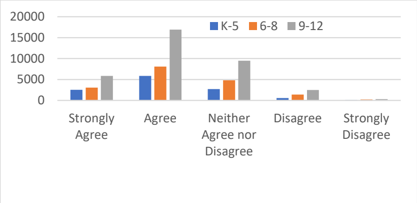

6 These videos did a good job representing the diversity of California school students. 64376

Strongly Agree and Agree combined total 54732

percentage 85%

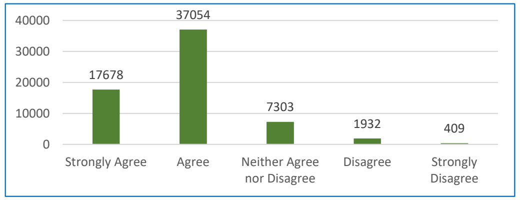

Strongly Agree and Agree combined total

k-5	6-8	9-12
88%	84%	84%

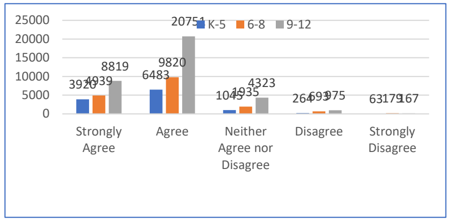

7 I feel the State of California is committed to supporting the social and emotional learning needs of teachers and students. 64376

Strongly Agree and Agree combined total 41576

percentage 65%

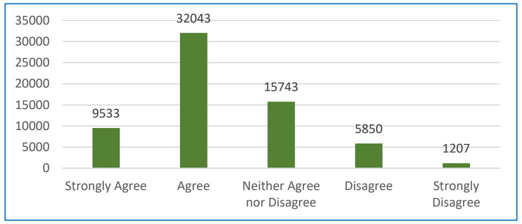

Strongly Agree and Agree combined total

k-5	6-8	9-12
63%	66%	64%

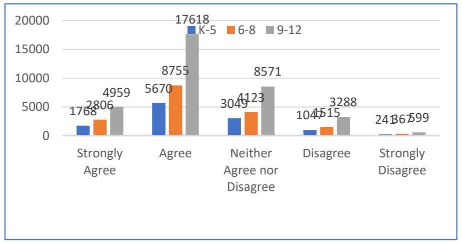

8 How likely are you to use the videos from this program in your classroom? 62646

Highly Likely and Likely combined total 51496

percentage 82%

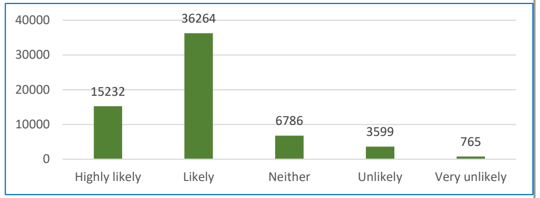

Strongly Agree and Agree combined total

k-5	6-8	9-12
84%	78%	84%

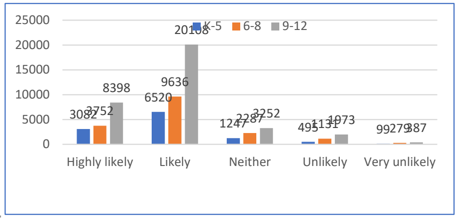

9 What would best motivate teachers like you to use the videos from this program in your classroom? 64376

c

Financial incentive and Integration into curriculum combined total 42781

percentage 66%

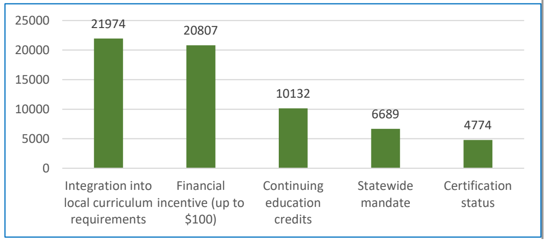

Strongly Agree and Agree combined total

k-5	6-8	9-12
66%	61%	69%

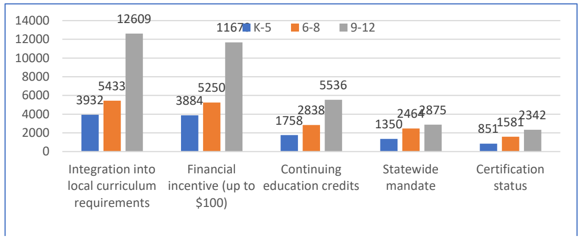

Supplement: S1 File — 2. Source data: Educator perspectives on the challenges of emotional health – before review of the HMTK program. 3: Source data: Educator perspectives after review of the HMTK program (PDF) [file pone.0305450.s001.pdf]
